# 9-(2,4-Dichloro­phen­yl)-3,3,6,6-tetra­methyl-3,4,5,6-tetra­hydro-9*H*-xanthene-1,8(2*H*,7*H*)-dione

**DOI:** 10.1107/S1600536810046702

**Published:** 2010-11-17

**Authors:** Hao Shi

**Affiliations:** a The College of Pharmaceutical Science, Zhejiang University of Technology, Hangzhou 310014, People’s Republic of China

## Abstract

The title compound, C_23_H_24_Cl_2_O_3_, was synthesized by reaction of 2,4-dichloro­benzaldehyde and 5,5-dimethyl­cyclo­hexane-1,3-dione in ethyl­ene glycol. The central ring of the xanthene moiety is almost planar (with an r.m.s. deviation of 0.0268 Å from the least-squares plane) while the two outer rings, in a *cis* arrangement, display envelope conformations. The ring of the 2,4-dichloro­phenyl substituent is nearly perpendicular [85.89 (4)°] to the xanthene ring system.

## Related literature

For related structures, see: Odabaşoğlu *et al.* (2008 ▶); Bigdeli *et al.* (2007 ▶); Tu *et al.* (2002 ▶, 2004 ▶); Jeyakanthan *et al.* (1999 ▶); Li *et al.* (2005 ▶); Shi *et al.* (1997 ▶). For applications of xanthene derivatives, see: Poupelin *et al.* (1978 ▶); Lambert *et al.* (1997 ▶); Menchen *et al.* (2003 ▶); Banerjee & Mukherjee (1981 ▶). For ring puckering parameters, see: Cremer & Pople (1975 ▶).
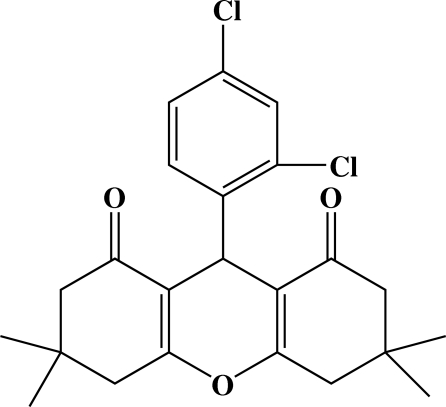


## Experimental

### 

#### Crystal data

C_23_H_24_Cl_2_O_3_
*M* *_r_* = 419.32
Monoclinic, 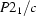

*a* = 9.8154 (10) Å
*b* = 19.833 (2) Å
*c* = 11.4441 (11) Å
β = 111.873 (2)°
*V* = 2067.4 (4) Å^3^
*Z* = 4
Mo *K*α radiation
μ = 0.34 mm^−1^
*T* = 296 K
0.20 × 0.10 × 0.10 mm

#### Data collection

Bruker APEX CCD diffractometer
Absorption correction: multi-scan (*SADABS*; Bruker, 1997 ▶) *T*_min_ = 0.936, *T*_max_ = 0.967
13137 measured reflections
4961 independent reflections
3331 reflections with *I* > 2σ(*I*)
*R*_int_ = 0.027

#### Refinement

*R*[*F* ^2^ > 2σ(*F* ^2^)] = 0.045
*wR*(*F* ^2^) = 0.124
*S* = 1.02
4961 reflections
257 parameters
H-atom parameters constrained
Δρ_max_ = 0.36 e Å^−3^
Δρ_min_ = −0.55 e Å^−3^

### 

Data collection: *SMART* (Bruker, 1997 ▶); cell refinement: *SAINT* (Bruker, 1997 ▶); data reduction: *SAINT*; program(s) used to solve structure: *SHELXS97* (Sheldrick, 2008 ▶); program(s) used to refine structure: *SHELXL97* (Sheldrick, 2008 ▶); molecular graphics: *ORTEP-3* (Farrugia, 1997 ▶); software used to prepare material for publication: *SHELXTL* (Sheldrick, 2008 ▶).

## Supplementary Material

Crystal structure: contains datablocks I, global. DOI: 10.1107/S1600536810046702/dn2622sup1.cif

Structure factors: contains datablocks I. DOI: 10.1107/S1600536810046702/dn2622Isup2.hkl

Additional supplementary materials:  crystallographic information; 3D view; checkCIF report

## Figures and Tables

**Fig. 1.**
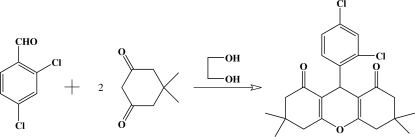
The preparation of the title compound.

**Fig. 2.**
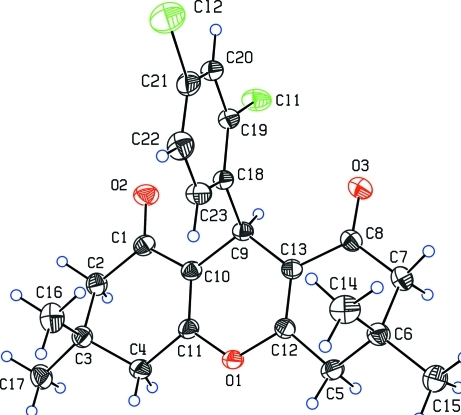
Structure of the title compound, showing 30% probability displacement ellipsoids with atomic numbering scheme.

## supplementary crystallographic information

### Comment

Xanthene-based compounds are biological importance and useful in drug discovery, such as anti-inflammatory effect (Poupelin *et al.*, 1978) and antiviral activity (Lambert *et al.*, 1997). Xanthenes are also an important class of organic compounds that find uses as dyes and fluorescent materials for visualization of bio-molecules and laser technologies due to their useful spectroscopic properties (Menchen *et al.*, 2003; Banerjee & Mukherjee, 1981). In view of the importance of the title compound, herein its crystal structure is reported.

The bond lengths and angles in the title molecule (Fig. 1) are comparable with those reported for related structures (Odabaşoğlu *et al.*, 2008; Bigdeli *et al.*, 2007; Tu *et al.*, 2002; Jeyakanthan *et al.*, 1999; Li *et al.*, 2005; Shi *et al.*, 1997; Tu *et al.*, 2004). The central pyran ring of the xanthene moiety is planar, and the two outer rings (ring A (C1—C4/C11/C10) and ring C (C5–C8/C13/C12)) are in envelope conformations, ring A with puckering parameters (Cremer & Pople, 1975) Q= 0.4511 (22) Å, θ= 125.45 (27)°, φ = 297.4 (3)° and ring C with puckering parameters Q= 0.4807 (22) Å, θ= 56.96 (25)°, φ = 58.0 (3)°. Rings A and C are in a *cis* conformation, atoms C3 and C6 displaced by 0.6311 (28) and 0.6684 (28) Å from the plane of the other ring atoms, respectively. Ring D (C18—C23) is, of course, planar. The dihedral angle between the least-squares plane of the xanthene ring and the benzene ring is 85.89 (4)°.

### Experimental

A mixture of 2,4-dichlorobenzaldehyde (5 mmol), 5,5-dimethyl-1,3- cyclohexanedione (10 mmol) and 15 ml of ethylene glycol was transferred into a flask connected with refluxing equipment. After stirring at 80°C for 1.5 h, the reaction mixture was cooled to room temperature and poured into 150 ml water, the precipitated product was filtered and recrystallized with ethanol to give the title compound. Crystals suitable for X-ray structure analysis were obtained by slow evaporation from a solution of methanol at room temperature.

### Refinement

All H atoms were placed in geometrical positions and constrained to ride on their parent atoms with C–H distances in the range 0.93–0.97 Å, They were treated as riding atoms, with *U*_iso_(H) = 1.5Ueq(C) for methyl H and 1.2Ueq(C) for other H atoms).

### Crystal data

**Table:**

C_23_H_24_Cl_2_O_3_	*F*(000) = 880
*M**_r_* = 419.32	*D*_x_ = 1.347 Mg m^−^^3^
Monoclinic, *P*2_1_/*c*	Mo *K*α radiation, λ = 0.71073 Å
Hall symbol: -P 2ybc	Cell parameters from 3489 reflections
*a* = 9.8154 (10) Å	θ = 2.2–27.1°
*b* = 19.833 (2) Å	µ = 0.34 mm^−^^1^
*c* = 11.4441 (11) Å	*T* = 296 K
β = 111.873 (2)°	Prism, colorless
*V* = 2067.4 (4) Å^3^	0.20 × 0.10 × 0.10 mm
*Z* = 4	

### Data collection

**Table:**

Bruker APEX CCD diffractometer	4961 independent reflections
Radiation source: fine-focus sealed tube	3331 reflections with *I* > 2σ(*I*)
graphite	*R*_int_ = 0.027
ω scans	θ_max_ = 28.2°, θ_min_ = 2.1°
Absorption correction: multi-scan (*SADABS*; Bruker, 1997)	*h* = −13→11
*T*_min_ = 0.936, *T*_max_ = 0.967	*k* = −25→26
13137 measured reflections	*l* = −13→14

### Refinement

**Table:**

Refinement on *F*^2^	Primary atom site location: structure-invariant direct methods
Least-squares matrix: full	Secondary atom site location: difference Fourier map
*R*[*F*^2^ > 2σ(*F*^2^)] = 0.045	Hydrogen site location: inferred from neighbouring sites
*wR*(*F*^2^) = 0.124	H-atom parameters constrained
*S* = 1.02	*w* = 1/[σ^2^(*F*_o_^2^) + (0.0573*P*)^2^ + 0.4531*P*] where *P* = (*F*_o_^2^ + 2*F*_c_^2^)/3
4961 reflections	(Δ/σ)_max_ < 0.001
257 parameters	Δρ_max_ = 0.36 e Å^−^^3^
0 restraints	Δρ_min_ = −0.55 e Å^−^^3^

### Special details

**Table:**

Geometry. All e.s.d.'s (except the e.s.d. in the dihedral angle between two l.s. planes) are estimated using the full covariance matrix. The cell e.s.d.'s are taken into account individually in the estimation of e.s.d.'s in distances, angles and torsion angles; correlations between e.s.d.'s in cell parameters are only used when they are defined by crystal symmetry. An approximate (isotropic) treatment of cell e.s.d.'s is used for estimating e.s.d.'s involving l.s. planes.
Refinement. Refinement of *F*^2^ against ALL reflections. The weighted *R*-factor *wR* and goodness of fit *S* are based on *F*^2^, conventional *R*-factors *R* are based on *F*, with *F* set to zero for negative *F*^2^. The threshold expression of *F*^2^ > σ(*F*^2^) is used only for calculating *R*-factors(gt) *etc*. and is not relevant to the choice of reflections for refinement. *R*-factors based on *F*^2^ are statistically about twice as large as those based on *F*, and *R*- factors based on ALL data will be even larger.

### Fractional atomic coordinates and isotropic or equivalent isotropic displacement parameters (Å^2^)

**Table:**

	*x*	*y*	*z*	*U*_iso_*/*U*_eq_	
Cl1	0.48123 (7)	0.49352 (3)	0.81227 (5)	0.05835 (18)	
Cl2	0.17633 (9)	0.68975 (4)	0.92235 (7)	0.0867 (3)	
O1	0.37359 (14)	0.63325 (7)	0.31501 (11)	0.0429 (3)	
O2	0.69826 (15)	0.60451 (8)	0.73455 (13)	0.0535 (4)	
O3	0.19747 (16)	0.47392 (7)	0.52949 (13)	0.0495 (4)	
C1	0.6678 (2)	0.63036 (10)	0.63116 (18)	0.0396 (4)	
C2	0.7753 (2)	0.67506 (10)	0.60270 (19)	0.0437 (5)	
H2A	0.8411	0.6468	0.5785	0.052*	
H2B	0.8341	0.6986	0.6793	0.052*	
C3	0.7073 (2)	0.72708 (10)	0.49949 (18)	0.0399 (4)	
C4	0.6005 (2)	0.68988 (11)	0.38461 (18)	0.0447 (5)	
H4A	0.5426	0.7227	0.3233	0.054*	
H4B	0.6561	0.6637	0.3461	0.054*	
C5	0.1471 (2)	0.57908 (11)	0.20690 (17)	0.0450 (5)	
H5A	0.1701	0.5446	0.1569	0.054*	
H5B	0.1296	0.6209	0.1598	0.054*	
C6	0.0085 (2)	0.55903 (10)	0.22869 (18)	0.0409 (4)	
C7	0.0469 (2)	0.49772 (10)	0.31638 (19)	0.0426 (4)	
H7A	−0.0365	0.4871	0.3390	0.051*	
H7B	0.0636	0.4594	0.2708	0.051*	
C8	0.1799 (2)	0.50728 (9)	0.43512 (18)	0.0369 (4)	
C9	0.42019 (19)	0.56963 (9)	0.55622 (16)	0.0336 (4)	
H9	0.4699	0.5268	0.5881	0.040*	
C10	0.52669 (18)	0.61624 (9)	0.52976 (16)	0.0345 (4)	
C11	0.50031 (19)	0.64432 (9)	0.41722 (17)	0.0368 (4)	
C12	0.27441 (19)	0.58782 (9)	0.32733 (17)	0.0363 (4)	
C13	0.29182 (19)	0.55638 (9)	0.43501 (17)	0.0342 (4)	
C14	−0.0441 (2)	0.61645 (11)	0.2899 (2)	0.0544 (6)	
H14A	0.0286	0.6256	0.3717	0.082*	
H14B	−0.1346	0.6038	0.2980	0.082*	
H14C	−0.0595	0.6561	0.2385	0.082*	
C15	−0.1116 (2)	0.54084 (13)	0.1031 (2)	0.0561 (6)	
H15A	−0.1238	0.5771	0.0444	0.084*	
H15B	−0.2022	0.5335	0.1151	0.084*	
H15C	−0.0845	0.5005	0.0707	0.084*	
C16	0.6252 (2)	0.78085 (11)	0.5432 (2)	0.0560 (6)	
H16A	0.5859	0.8140	0.4782	0.084*	
H16B	0.6917	0.8021	0.6181	0.084*	
H16C	0.5465	0.7601	0.5608	0.084*	
C17	0.8269 (2)	0.76069 (11)	0.4648 (2)	0.0512 (5)	
H17A	0.8768	0.7272	0.4351	0.077*	
H17B	0.8958	0.7826	0.5377	0.077*	
H17C	0.7833	0.7935	0.3998	0.077*	
C18	0.36693 (19)	0.59939 (9)	0.65453 (16)	0.0342 (4)	
C19	0.3866 (2)	0.56903 (10)	0.76900 (17)	0.0384 (4)	
C20	0.3308 (2)	0.59704 (11)	0.85255 (18)	0.0453 (5)	
H20	0.3460	0.5763	0.9293	0.054*	
C21	0.2526 (2)	0.65597 (11)	0.8201 (2)	0.0492 (5)	
C22	0.2324 (2)	0.68823 (11)	0.7101 (2)	0.0521 (5)	
H22	0.1810	0.7287	0.6903	0.063*	
C23	0.2899 (2)	0.65966 (10)	0.62823 (19)	0.0441 (5)	
H23	0.2765	0.6816	0.5529	0.053*	

### Atomic displacement parameters (Å^2^)

**Table:**

	*U*^11^	*U*^22^	*U*^33^	*U*^12^	*U*^13^	*U*^23^
Cl1	0.0722 (4)	0.0568 (3)	0.0467 (3)	0.0159 (3)	0.0229 (3)	0.0185 (2)
Cl2	0.1098 (6)	0.0919 (5)	0.0885 (5)	−0.0067 (4)	0.0717 (5)	−0.0323 (4)
O1	0.0408 (7)	0.0571 (8)	0.0295 (7)	−0.0146 (6)	0.0117 (6)	0.0025 (6)
O2	0.0466 (8)	0.0693 (10)	0.0385 (8)	−0.0043 (7)	0.0090 (6)	0.0099 (7)
O3	0.0550 (8)	0.0487 (8)	0.0469 (9)	−0.0060 (7)	0.0217 (7)	0.0081 (7)
C1	0.0378 (10)	0.0441 (11)	0.0370 (10)	0.0025 (8)	0.0139 (8)	−0.0023 (8)
C2	0.0358 (10)	0.0477 (11)	0.0460 (12)	−0.0039 (8)	0.0136 (9)	−0.0014 (9)
C3	0.0368 (9)	0.0430 (10)	0.0417 (11)	−0.0042 (8)	0.0165 (8)	−0.0025 (8)
C4	0.0445 (11)	0.0556 (12)	0.0362 (10)	−0.0118 (9)	0.0178 (9)	0.0004 (9)
C5	0.0427 (11)	0.0608 (13)	0.0305 (10)	−0.0103 (9)	0.0125 (8)	−0.0009 (9)
C6	0.0366 (9)	0.0465 (11)	0.0399 (10)	−0.0028 (8)	0.0145 (8)	0.0024 (8)
C7	0.0381 (10)	0.0425 (11)	0.0492 (12)	−0.0048 (8)	0.0187 (9)	0.0006 (9)
C8	0.0402 (10)	0.0335 (9)	0.0424 (11)	0.0010 (8)	0.0215 (8)	−0.0003 (8)
C9	0.0362 (9)	0.0351 (9)	0.0323 (9)	0.0024 (7)	0.0160 (8)	0.0009 (7)
C10	0.0331 (9)	0.0401 (10)	0.0320 (9)	0.0000 (7)	0.0143 (8)	−0.0012 (7)
C11	0.0340 (9)	0.0450 (10)	0.0324 (9)	−0.0049 (8)	0.0136 (8)	−0.0034 (8)
C12	0.0358 (9)	0.0415 (10)	0.0352 (10)	−0.0054 (8)	0.0174 (8)	−0.0026 (8)
C13	0.0352 (9)	0.0366 (9)	0.0341 (9)	0.0000 (7)	0.0167 (8)	−0.0017 (7)
C14	0.0524 (12)	0.0540 (13)	0.0575 (14)	0.0137 (10)	0.0210 (11)	0.0055 (10)
C15	0.0432 (11)	0.0692 (15)	0.0485 (13)	−0.0118 (11)	0.0084 (10)	0.0017 (11)
C16	0.0478 (12)	0.0498 (12)	0.0728 (16)	−0.0003 (10)	0.0252 (11)	−0.0086 (11)
C17	0.0466 (11)	0.0529 (12)	0.0578 (14)	−0.0114 (10)	0.0236 (10)	−0.0036 (10)
C18	0.0356 (9)	0.0382 (10)	0.0303 (9)	−0.0028 (7)	0.0139 (7)	−0.0010 (7)
C19	0.0401 (10)	0.0422 (10)	0.0318 (9)	−0.0035 (8)	0.0120 (8)	0.0005 (8)
C20	0.0506 (11)	0.0550 (12)	0.0338 (10)	−0.0132 (10)	0.0198 (9)	−0.0049 (9)
C21	0.0542 (12)	0.0544 (13)	0.0502 (13)	−0.0108 (10)	0.0322 (11)	−0.0170 (10)
C22	0.0553 (12)	0.0459 (12)	0.0600 (14)	0.0058 (10)	0.0270 (11)	−0.0067 (10)
C23	0.0521 (11)	0.0431 (11)	0.0396 (11)	0.0056 (9)	0.0202 (9)	0.0033 (8)

### Geometric parameters (Å, °)

**Table:**

Cl1—C19	1.734 (2)	C9—C10	1.508 (2)
Cl2—C21	1.741 (2)	C9—C13	1.509 (2)
O1—C11	1.371 (2)	C9—C18	1.525 (2)
O1—C12	1.372 (2)	C9—H9	0.9800
O2—C1	1.220 (2)	C10—C11	1.337 (2)
O3—C8	1.223 (2)	C12—C13	1.334 (2)
C1—C10	1.465 (3)	C14—H14A	0.9600
C1—C2	1.504 (3)	C14—H14B	0.9600
C2—C3	1.522 (3)	C14—H14C	0.9600
C2—H2A	0.9700	C15—H15A	0.9600
C2—H2B	0.9700	C15—H15B	0.9600
C3—C17	1.526 (3)	C15—H15C	0.9600
C3—C16	1.528 (3)	C16—H16A	0.9600
C3—C4	1.531 (3)	C16—H16B	0.9600
C4—C11	1.482 (3)	C16—H16C	0.9600
C4—H4A	0.9700	C17—H17A	0.9600
C4—H4B	0.9700	C17—H17B	0.9600
C5—C12	1.486 (3)	C17—H17C	0.9600
C5—C6	1.525 (3)	C18—C23	1.386 (3)
C5—H5A	0.9700	C18—C19	1.388 (2)
C5—H5B	0.9700	C19—C20	1.384 (3)
C6—C15	1.524 (3)	C20—C21	1.371 (3)
C6—C14	1.524 (3)	C20—H20	0.9300
C6—C7	1.531 (3)	C21—C22	1.360 (3)
C7—C8	1.505 (3)	C22—C23	1.383 (3)
C7—H7A	0.9700	C22—H22	0.9300
C7—H7B	0.9700	C23—H23	0.9300
C8—C13	1.468 (2)		
			
C11—O1—C12	118.07 (14)	C10—C11—O1	122.95 (16)
O2—C1—C10	120.39 (18)	C10—C11—C4	126.02 (16)
O2—C1—C2	121.29 (17)	O1—C11—C4	111.03 (15)
C10—C1—C2	118.28 (17)	C13—C12—O1	123.39 (16)
C1—C2—C3	115.27 (15)	C13—C12—C5	125.20 (17)
C1—C2—H2A	108.5	O1—C12—C5	111.40 (15)
C3—C2—H2A	108.5	C12—C13—C8	118.18 (16)
C1—C2—H2B	108.5	C12—C13—C9	122.91 (16)
C3—C2—H2B	108.5	C8—C13—C9	118.91 (15)
H2A—C2—H2B	107.5	C6—C14—H14A	109.5
C2—C3—C17	109.89 (16)	C6—C14—H14B	109.5
C2—C3—C16	110.57 (17)	H14A—C14—H14B	109.5
C17—C3—C16	109.31 (17)	C6—C14—H14C	109.5
C2—C3—C4	107.56 (16)	H14A—C14—H14C	109.5
C17—C3—C4	109.51 (16)	H14B—C14—H14C	109.5
C16—C3—C4	109.97 (16)	C6—C15—H15A	109.5
C11—C4—C3	112.57 (16)	C6—C15—H15B	109.5
C11—C4—H4A	109.1	H15A—C15—H15B	109.5
C3—C4—H4A	109.1	C6—C15—H15C	109.5
C11—C4—H4B	109.1	H15A—C15—H15C	109.5
C3—C4—H4B	109.1	H15B—C15—H15C	109.5
H4A—C4—H4B	107.8	C3—C16—H16A	109.5
C12—C5—C6	111.83 (15)	C3—C16—H16B	109.5
C12—C5—H5A	109.2	H16A—C16—H16B	109.5
C6—C5—H5A	109.2	C3—C16—H16C	109.5
C12—C5—H5B	109.2	H16A—C16—H16C	109.5
C6—C5—H5B	109.2	H16B—C16—H16C	109.5
H5A—C5—H5B	107.9	C3—C17—H17A	109.5
C15—C6—C14	109.56 (17)	C3—C17—H17B	109.5
C15—C6—C5	109.34 (16)	H17A—C17—H17B	109.5
C14—C6—C5	110.85 (17)	C3—C17—H17C	109.5
C15—C6—C7	110.20 (17)	H17A—C17—H17C	109.5
C14—C6—C7	109.58 (17)	H17B—C17—H17C	109.5
C5—C6—C7	107.28 (16)	C23—C18—C19	116.87 (17)
C8—C7—C6	114.26 (16)	C23—C18—C9	118.85 (16)
C8—C7—H7A	108.7	C19—C18—C9	124.25 (16)
C6—C7—H7A	108.7	C20—C19—C18	121.75 (18)
C8—C7—H7B	108.7	C20—C19—Cl1	117.22 (15)
C6—C7—H7B	108.7	C18—C19—Cl1	121.03 (15)
H7A—C7—H7B	107.6	C21—C20—C19	118.76 (19)
O3—C8—C13	120.01 (17)	C21—C20—H20	120.6
O3—C8—C7	121.26 (17)	C19—C20—H20	120.6
C13—C8—C7	118.70 (16)	C22—C21—C20	121.69 (19)
C10—C9—C13	109.02 (14)	C22—C21—Cl2	119.39 (18)
C10—C9—C18	111.39 (14)	C20—C21—Cl2	118.91 (17)
C13—C9—C18	110.31 (14)	C21—C22—C23	118.6 (2)
C10—C9—H9	108.7	C21—C22—H22	120.7
C13—C9—H9	108.7	C23—C22—H22	120.7
C18—C9—H9	108.7	C22—C23—C18	122.25 (19)
C11—C10—C1	118.05 (17)	C22—C23—H23	118.9
C11—C10—C9	123.28 (16)	C18—C23—H23	118.9
C1—C10—C9	118.66 (16)		
